# *Mycobacterium genavense *as a cause of subacute pneumonia in patients with severe cellular immunodeficiency

**DOI:** 10.1186/1471-2334-11-311

**Published:** 2011-11-05

**Authors:** Blandine Rammaert, Louis-Jean Couderc, Elisabeth Rivaud, Patrick Honderlick, David Zucman, Marie-France Mamzer, Pierre Cahen, Emmanuel Bille, Marc Lecuit, Olivier Lortholary, Emilie Catherinot

**Affiliations:** 1 Université Paris-Descartes, Hôpital Necker-Enfants Malades, Service des Maladies Infectieuses et Tropicales, Centre d'Infectiologie Necker-Pasteur, Paris, France; 2 Université Versailles-Saint Quentin, Hôpital Foch, Service de Pneumologie, Suresnes, France; 3 Hôpital Foch, Service de Microbiologie, Suresnes, France; 4 Hôpital Foch, Service de Médecine Interne, Suresnes, France; 5 Université Paris-Descartes, Hôpital Necker-Enfants malades, Service de Transplantation Rénale, Paris, France; 6 Université Paris-Descartes, Hôpital Necker-Enfants malades, Service de Microbiologie, Paris, France; 7 Institut Pasteur, Groupe Microorganismes et barrières de l'hôte, Inserm avenir U604, Paris, France; 8 Institut Pasteur, Unité de Mycologie Moléculaire, Centre National de Référence Mycologie et Antifongiques, CNRS URA3012, Paris, France

## Abstract

**Background:**

*Mycobacterium genavense *is a rare nontuberculous mycobacteria (NTM). Human infections are mostly disseminated in the setting of the AIDS epidemic or the use of aggressive immunosuppressive treatments. *M. genavense *culture is fastidious, requiring supplemented media. Pulmonary involvement rarely occurs as a primary localization.

**Cases presentation:**

We report here two patients with pneumonia as the predominant manifestation of *M. genavense *infection: one kidney transplanted patient and one HIV-infected patient. Both patients were initially treated with anti-tuberculous drugs before the identification of *M. genavense *on sputum or broncho-alveolar lavage fluid culture. A four-drug regimen including clarithromycin and rifabutin was started. Gamma interferon has been helpful in addition to antimycobacterial treatment for one patient.

**Conclusion:**

Clinicians should be aware that *M. genavense *could be the etiologic agent of sub-acute pneumonia mimicking tuberculosis in patients with cellular immunodeficiency status.

## Background

Host defenses against mycobacteria need an immune cellular response (Th1/Th17) activated by cytokines and chemokines produced by innate immunity cells [1]. Consequently, non-tuberculous mycobacteria (NTM) could cause life threatening infection in immunocompromised patients with profound cellular immune deficiency in particular those who are HIV-infected [2]. *M. genavense*, is a fastidious growing mycobacteria found in water [3] with birds and pets being occasionally infected [4]. Human infections were initially described in HIV-infected patients [5]. Thereafter, few cases have been observed in non-HIV immunocompromised hosts including only two reports in solid organ transplant recipients [6,7]. *M. genavense *infection preferentially involves bowel and abdominal lymph nodes, a fact being ascribed to the presumed digestive contamination. Mortality of patients with disseminated *M. genavense *infection is high, ranging from 44% to 71% in 2 series [8,9]. Herein we report two cases of *M. genavense *infected patients with pulmonary involvement as the main clinical manifestation, one kidney transplant recipient and one HIV-infected patient.

## Cases presentation

### Case one

**Figure 1:**
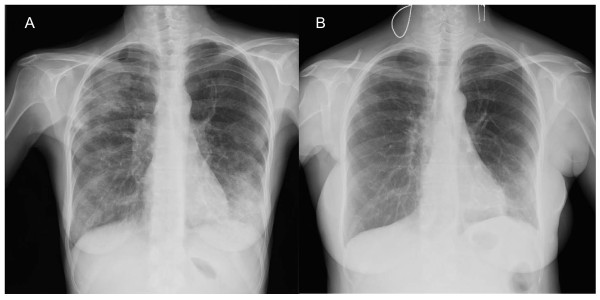
**Chest radiograph of case 1**. A: At presentation, diffuse bilateral pulmonary infiltrates predominant to upper right and lower left lobes. B: Major improvement after 2 years of treatment.

**Figure 2:**
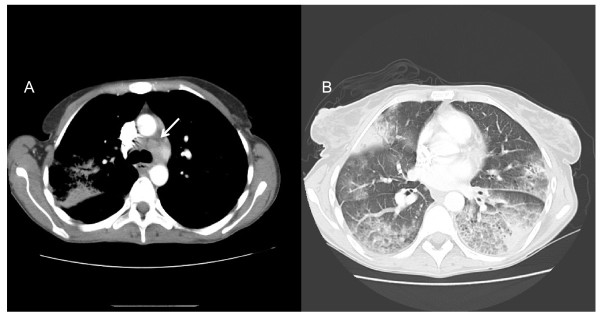
**Thoracic CT Scan of case 1 at presentation**. A: Mediastinal lymphadenopathies. B: Bilateral ground glass opacities and areas of consolidation.

A 43-year-old woman, of Moroccan origin, was admitted in August 2005 for seizure, an 8 kg loss of body-weight, slowly increasing dyspnea, productive cough, and fever lasting for 4 months. She had been known to be infected by HIV since 1996, but lost to follow-up. Physical examination revealed left lower limb palsy, pulmonary ronchi, hepatomegaly and oropharyngeal candidiasis. Cerebral computed tomography (CT) imaging demonstrated multiple abscesses. Cerebral toxoplasmosis was suspected and treatment with pyrimethamine and sulfadiazine was initiated. Biological results were: C-Reactive Protein (CRP) 19 mg/L, leukocyte count 3940/mm^3^, hemoglobin 11,1 g/dL, aspartate aminotransferase 88 U/L, alanine aminotransferase 40 U/L, gamma glutamyl transferase 310 U/L, alkaline phosphatases 210 U/L, lactico deshydrogenase 268 U/L. Her peripheral blood CD4+ cell count was 110/mm^3 ^and HIV viral load was 6.39 log copies/mL. Serological tests for hepatitis B and C were positive. A chest radiograph demonstrated diffuse bilateral pulmonary infiltrates (Figure 1A). The thoracic CT-scan showed mediastinal lymphadenopathies, numerous diffuse pulmonary nodular infiltrates with a cavitation in the left upper lobe (Figure 2). Bronchial endoscopy did not show abnormalities. Three sputa were smear positive for acid-fast bacilli (AFB). Broncho-alveolar lavage (BAL) fluid cytological analysis confirmed the presence of AFB but was negative for other microorganisms. Isoniazid, rifampicin and pyrazinamide were therefore started. Despite the anti-tuberculous regimen, fever persisted and respiratory symptoms progressively worsened requiring continuous nasal oxygenotherapy. After 10 weeks, mycobacterial culture from the respiratory specimen grew *M. genavense*. The initial anti-tuberculosis treatment was changed for a multidrug regimen with moxifloxacin (400 mg/d), clarithromycin (500 mg b.i.d), ethambutol (20 mg/kg/d) and amikacin (15 mg/kg/d, during 3 weeks) and HAART (tenofovir, emtricitabine, efavirenz) were introduced. Clarithromycin was increased to 1000 mg b.i.d after 2 weeks of treatment because the treatment was well-tolerated and optimal treatment recommendations were sparse. HIV viral load decreased to 3 log copies/mL after 1 month without increase in CD4+ count (60 cells/mm^3^). Despite 3 months of this antimycobacterial treatment, the patient's clinical course worsened and infiltrates in the upper lobes on chest X-ray increased. A second bronchoscopy was performed and BAL fluid cytologic analysis showed the persistence of 20 to 30 AFB/field, which were again identified as *M. genavense*, without other pathogens. Then gamma interferon (IFN-γ) was started (50 μg/m^2 ^sub-cutaneously 3-times a week). The HAART therapy was continued with undetectable viral load but a persistent CD4 lymphopenia (50/mm^3^). The patient's general condition gradually improved within 1 month of IFN-γ treatment with oxygen weaning, radiological improvement and negative respiratory specimens mycobacterial culture. The CD4 cell count reached 300/mm^3 ^after twelve months of antimycobacterial treatment, so IFN-γ was withdrawn. The anti-mycobacterial treatment was withdrawn after 2 years. We decided to continue the treatment more than 6 months after immune reconstitution and 12 months after negative culture of respiratory specimen because chest radiograph demonstrated continuous regression of pulmonary infiltrate until 24 months, with minimal sequelae at the end of treatment (Figure 1B). At the last medical visit in June 2011, the patient remained in good health, without relapse of the mycobacterial disease.

### Case two

**Figure 3:**
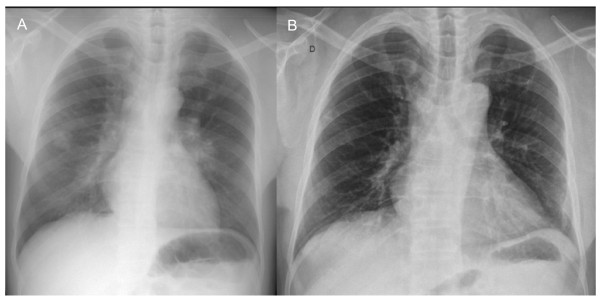
**Chest radiograph of case 2**. A: At presentation, alveolar consolidations predominant to right lung and parahilar left area. B: Major improvement after 2 years of treatment.

**Figure 4:**
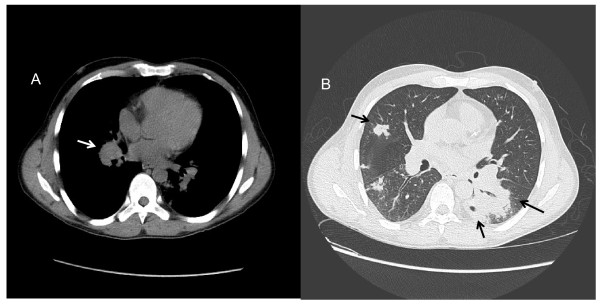
**Thoracic CT Scan of case 2 at presentation**. A: Right hilar lymphadenopathy. B: Multiple nodules in right lung. Areas of consolidation with cavitation in left lower lobe.

A 44 year-old renal transplant Guinean patient was admitted in September 2008 for fever, weight loss and non-productive cough worsening gradually for one month. He had undergone renal transplantation 3 years earlier because of focal segmentary hyalinosis. His immunosuppressive regimen included mycophenolate mofetil 1500 mg/d, prednisone 10 mg/d and tacrolimus 6 mg/d without any modification since. Blood test results were: CRP 28 mg/L; white blood cells 11300/mm^3^; hemoglobin 10.9 g/dL. CD4+ lymphocyte count was 230 cells/mm^3^. Serological tests for HIV-1 and HIV-2 were negative. Chest radiograph (Figure 3A) and thoracic CT-scan showed areas of alveolar consolidation and cavitations in both lungs, multiple mediastinal and enlarged hilar lymph nodes (Figure 4). Bronchial endoscopy revealed complete stenosis of the left lower bronchi and corresponding histological analysis revealed an epithelioid cell granuloma without necrosis. The BAL fluid analysis revealed 670,000 cells/ml with 45% neutrophils, 27% macrophages, 27% lymphocytes, and 1% eosinophils. The microbiological studies did not provide evidence for bacterial, fungal or viral infection neither in BAL fluid nor in biopsy specimens. The patient then underwent an open lung surgical biopsy. Lung histological analysis revealed multiple parenchymal epitheloid gigantocellular granulomas with some eosinophilic necrosis. An anti-tuberculous regimen (isoniazid, rifampin, ethambutol) was started. Amplification of the rDNA 16S allowing detection of a broad range of bacteria, the IS6110 of *Mycobacterium tuberculosis *and the 16S-23S rDNA gene spacer targeting all mycobacteria, were negative on pulmonary biopsy. After surgery, the patient developed abdominal pain with no intestinal transit. Abdominal CT scan revealed two mesenteric necrotic lymphadenopathies, colic dilatation, bowel-wall thickening, and gastric stasis. A colonoscopy showed apthoid ulcerations from the caecum to the transversal colon. Biopsies disclosed ulcerative colitis with epithelioid cell granulomas. An esogastric endoscopic examination was normal. A few days later, one in three post fibroscopic sputum samples grew in one month (10 days after the thoracic surgery) on Mycobacteria Growth Indicator Tube (MGIT) liquid medium. *M. genavense *was identified by INNO-LiPA probe identification on the product of the 16S-23S rDNA gene spacer region amplification. Anti-mycobacterial therapy was thus modified for clarithromycin (500 mg b.i.d), moxifloxacin (400 mg/d), rifabutin (5 mg/kg/d) and ethambutol (15 mg/kg/d). The immunosuppressive regimen was tapered with withdrawal of mycophenolate, tacrolimus was replaced by cyclosporin A, so the daily dose of prednisone was increased to 20 mg. Blood and bone marrow cultures were negative. Bone scan showed no additional localization. Intestinal malabsorption and anorexia required prolonged parenteral nutrition. After 3 months of treatment, the patient's condition gradually improved; fever, respiratory and digestive symptoms had resolved. The patient was no longer able to produce sputum for mycobacterial culture. Colonoscopic control after 2 months of treatment showed the regression of ulcerations. After 6 months, lung lesions and mesenteric lymphadenopathies had decreased on thoracic and abdominal CT scan. At the last medical visit, in October 2010, abdominal CT scan showed resolution of adenopathies. On thoracic CT scan, pulmonary consolidations had resolved. One right hilar lymph node (13 mm) remained unchanged. The antimycobacterial treatment was continued because of the sustained immunosuppressive regimen for chronic graft rejection.

## Conclusions

We here emphasize the predominant thoracic involvement that revealed severe *M. genavense *infection in two patients with cellular immune deficiency. Secondary dissemination occurred in one case. *M. genavense *infection has previously been reported in only two solid organ (kidney and heart) transplanted patients [10,11]. Both had intestinal involvement leading to secondary dissemination and death in one case [10]. In HIV-infected patients, disseminated *M. genavense *infection associates weight loss, fever, anemia, digestive manifestations and hepatosplenomegaly [9,12,13]. Pulmonary symptoms such as cough and dyspnea are rarely at the forefront. A retrospective multicenter French study of 25 patients with *M. genavense *infection (of whom 20 were HIV positive) reported five patients with pulmonary symptoms in combination with extra-respiratory symptoms in all cases [8]. However, *M. genavense *remains a rare cause of NTM infection. In the Microbiology Department of Foch Hospital, Suresnes, our two cases were the only two patients with *M. genavense *isolation among 176 patients with NTM isolated from clinical specimens between 2005 and 2008. The other mycobacteria identified were: *Mycobacterium avium complex*: 56; *M. gordonae*: 42; *M. xenopi*: 37; *M. kansasii: *21; *M. fortuitum*: 8; *M. abscessus complex *or *M. chelonae*: 7; *M. celatum*: 1; non-identified: 2.

*M. genavense *can be cultured from stools, sputum, urine and blood samples and tissue biopsy [14]. However, culture of *M. genavense *is fastidious, making microbiological diagnosis frequently difficult, requiring mycobactin J supplementation for optimal recovery on culture. Culture must be prolonged up to 4 months with a median positive culture time of 43 days (min. 10 days- max. 6 months) depending on the culture medium [5,9,11,13,15-18]. Our observations are in agreement with these findings, In our first case, diagnosis was delayed because the time to positive culture was 70 days.

Molecular tools such as polymerase chain reaction (PCR) are of interest in two ways before cultures become positive. Firstly, *M. genavense *can eventually be detected on clinical specimens with PCR [19-21]. Secondly, a negative result for *M. tuberculosis *PCR on a positive AFB smear specimen is useful to exclude tuberculosis earlier. In case one, NTM treatment would have been considered if *M. tuberculosis *PCR had been performed on sputum. Furthermore, we think that *M. genavense *should be envisaged in immunocompromised patients with mycobacterial pulmonary disease and digestive involvement or negative mycobacterial culture lasting for more than one month.

Infection control requires decrease of the immunosuppressive regimen in patients who receive such treatment in combination with appropriate antimycobacterial treatment. Human and mouse models have pointed out the role of CD4+ and CD8+ T lymphocytes in *M. genavense *infection [22,23]. Explaining the crucial role of T lymphocytes, the bacterial load was significantly higher in organs from athymic mice compared to normal mice [23]. During HIV infection, the risk of *M. genavense *disease is correlated with CD4 cell count. Indeed *M. genavense *infection occurs in patients with profound CD4+ lymphopenia (50 cells/mm^3 ^or less). However, CD4+ cell count is not a reliable marker of immunosuppression in non-HIV patients receiving immunosuppressive treatment. For example, *Pneumocystis *pneumonia is frequently observed in organ transplanted patients with more than 400 CD4+ cells/mm^3 ^[24]. In our patient, the CD4+ T cell count was moderately diminished (230/mm^3^). Thus, *M. genavense *infection may occur in non-HIV patients under an immunosuppressive regimen with a much higher CD4+ cell count than seen in HIV-infected patients.

The optimal anti-mycobacterial therapy in *M. genavense *infection is unknown. The high effectiveness of clarithromycin and rifabutin has been demonstrated in a murine model [25]. Multidrug therapies including clarithromycin are recommended [26]. Even with appropriate treatment, clinical evolution is long before resolution of symptoms, and patients have to be treated for at least 12 to 24 months [26]. As recommended in ATS/IDSA guidelines for *Mycobacterium avium intracellulare *infection, treatment was continued 12 months after immune restoration was achieved for Case 1 [26]. Concerning Case 2, antimycobacterial treatment cannot be withdrawn because of reinforcement of immunosuppressive therapy.

In the case of our first patient, despite HAART introduction and appropriate anti-mycobacterial treatment, the infection was not controlled. The administration of IFN-γ was temporally associated with a significant clinical improvement and bacteriological clearance of infection. IFN-γ is essential for the control of *M. genavense*. Indeed, mice deficient for IFN-γ are unable to clear *M. genavense *infection [27]. In HIV-infected individuals the monocytes-macrophages phagocytic function is impaired. IFN-γ can restore deficient functions of HIV-infected macrophages and has already been used in the management of *M. avium intracellulare *complex infection [28]. The classic pathway of IFN-γ-dependent activation of macrophages by T helper 1-type responses is a well-established feature of immune response to infection with intracellular pathogens, such as *M. tuberculosis *and NTM [22].

Finally clinicians should be aware 1) That the main clinical manifestation of *M. genavense *infection may be a slowly progressive pneumonia mimicking tuberculosis in patients with cellular immunodeficiency; 2) Thus, this diagnosis should be evoked in an immunosuppressed patient who receives empirical anti-tuberculous therapy without improvement; 3) Extra-thoracic manifestations can be absent; 4) the microbiological diagnosis requires prolonged cultures and may be helped by PCR analysis of bronchial and lung specimens; 5) IFN-γ may be useful to control *M. genavense *pneumonia in addition to the anti-mycobacterial regimen; 6) CD4+ T cell count is not a reliable marker of the risk of *M. genavense *infection in non-HIV imunocompromised patients.

## Abbreviations

Th: T helper lymphocyte; HAART: Highly active anti-retroviral therapy; AFB: Acid fast bacilli; BAL: broncho-alveolar lavage; HIV: Human immunodeficiency virus; PCR: Polymerase chain reaction; NTM: Non tuberculous mycobacteria; IFN-γ: Interferon-gamma.

## Competing interests

The authors declare that they have no competing interests.

## Consent

Written informed consent was obtained from the patient for publication of this case report and accompanying images. A copy of the written consent is available for review by the Editor-in-Chief of this journal

## Authors' contributions

BR, EC, OL, LJC made substantial contributions to conception and design. ER, PH, DZ, MFM, PC, ML participated in acquisition of data. BR, EC drafted the manuscript. OL, LJC, ER, PH, DZ, MFM, PC, ML revised it critically. All authors read and approved the final manuscript.

## Pre-publication history

The pre-publication history for this paper can be accessed here:

http://www.biomedcentral.com/1471-2334/11/311/prepub

## References

[B1] Mortellaro A Robinson L Ricciardi-Castagnoli P Spotlight on Mycobacteria and dendritic cells: will novel targets to fight tuberculosis emerge? EMBO Mol Med 2009 1 1 19 29 10.1002/emmm.200900008 20049700 PMC3378112

[B2] Glassroth J Pulmonary disease due to nontuberculous mycobacteria Chest 2008 133 1 243 251 10.1378/chest.07-0358 18187749

[B3] Hillebrand-Haverkort ME Kolk AH Kox LF Ten Velden JJ Ten Veen JH Generalized mycobacterium genavense infection in HIV-infected patients: detection of the mycobacterium in hospital tap water Scand J Infect Dis 1999 31 1 63 68 10.1080/00365549950161907 10381220

[B4] Manarolla G Liandris E Pisoni G Sassera D Grilli G Gallazzi D Sironi G Moroni P Piccinini R Rampin T Avian mycobacteriosis in companion birds: 20-year survey Vet Microbiol 2009 133 4 323 327 10.1016/j.vetmic.2008.07.017 18789612

[B5] Bessesen MT Shlay J Stone-Venohr B Cohn DL Reves RR Disseminated Mycobacterium genavense infection: clinical and microbiological features and response to therapy AIDS 1993 7 10 1357 1361 10.1097/00002030-199310000-00009 8267909

[B6] Lu KJ Grigg A Leslie D Finlay M Sasadeusz J Mycobacterium genavense duodenitis following allogeneic peripheral blood stem cell transplantation Transpl Infect Dis 2009 11 6 534 536 10.1111/j.1399-3062.2009.00431.x 19656345

[B7] Bogdan C Kern P Richter E Tannapfel A Rusch-Gerdes S Kirchner T Solbach W Systemic infection with Mycobacterium genavense following immunosuppressive therapy in a patient who was seronegative for human immunodeficiency virus Clin Infect Dis 1997 24 6 1245 1247 10.1086/513634 9195092

[B8] Charles P Lortholary O Dechartres A Doustar F Viard J-P Lecuit M Gutierrez MC Group TFMgS Mycobacterium genavense Infections: a retrospective Multicenter Study between 1996 and 2007 in France Medicine 2011 90 4 223 30 10.1097/MD.0b013e318225ab89 21694645

[B9] Tortoli E Brunello F Cagni AE Colombrita D Dionisio D Grisendi L Manfrin V Moroni M Passerini Tosi C Pinsi G Mycobacterium genavense in AIDS patients, report of 24 cases in Italy and review of the literature Eur J Epidemiol 1998 14 3 219 224 10.1023/A:1007401305708 9663512

[B10] Nurmohamed S Weenink A Moeniralam H Visser C Bemelman F Hyperammonemia in generalized Mycobacterium genavense infection after renal transplantation Am J Transplant 2007 7 3 722 723 10.1111/j.1600-6143.2006.01680.x 17250553

[B11] de Lastours V Guillemain R Mainardi JL Aubert A Chevalier P Lefort A Podglajen I Early diagnosis of disseminated Mycobacterium genavense infection Emerg Infect Dis 2008 14 2 346 347 10.3201/eid1402.070901 18258141 PMC2600216

[B12] Pechere M Opravil M Wald A Chave JP Bessesen M Sievers A Hein R von Overbeck J Clark RA Tortoli E Clinical and epidemiologic features of infection with Mycobacterium genavense. Swiss HIV Cohort Study Arch Intern Med 1995 155 4 400 404 10.1001/archinte.155.4.400 7848023

[B13] Thomsen VO Dragsted UB Bauer J Fuursted K Lundgren J Disseminated infection with Mycobacterium genavense: a challenge to physicians and mycobacteriologists J Clin Microbiol 1999 37 12 3901 3905 10565904 10.1128/jcm.37.12.3901-3905.1999 PMC85841

[B14] Dumonceau JM Van Gossum A Adler M Van Vooren JP Fonteyne PA De Beenhouwer H Portaels F Detection of fastidious mycobacteria in human intestines by the polymerase chain reaction Eur J Clin Microbiol Infect Dis 1997 16 5 358 363 10.1007/BF01726363 9228475

[B15] Delpire P Farber CM Portaels F Struelens M Clevenbergh P Dargent JL Delpace J Mehdi A Van Vooren JP Splenectomy in a patients with AIDS, generalized Mycobacterium genavense infection and severe pancytopenia Tuber Lung Dis 1996 77 6 569 570 10.1016/S0962-8479(96)90059-3 9039454

[B16] Krebs T Zimmerli S Bodmer T Lammle B Mycobacterium genavense infection in a patient with long-standing chronic lymphocytic leukaemia J Intern Med 2000 248 4 343 348 10.1046/j.1365-2796.2000.00730.x 11086646

[B17] Leautez S Boutoille D Bemer-Melchior P Ponge T Raffi F Localized Mycobacterium genavense soft tissue infection in an immunodeficient HIV-negative patient Eur J Clin Microbiol Infect Dis 2000 19 1 51 52 10.1007/s100960050010 10706181

[B18] Trueba F Fabre M Saint-Blancard P Rapid identification of Mycobacterium genavense with a new commercially available molecular test, INNO-LiPA MYCOBACTERIA v2 J Clin Microbiol 2004 42 9 4403 4404 10.1128/JCM.42.9.4403-4404.2004 15365056 PMC516359

[B19] Ninet B Rutschmann O Burkhardt K Metral C Borisch B Hirschel B Detection of mycobacterial nucleic acids by polymerase chain reaction in fixed tissue specimens of patients with human immunodeficiency virus infection. Swiss HIV Cohort Study Diagn Mol Pathol 1999 8 3 145 151 10.1097/00019606-199909000-00007 10565686

[B20] Chevrier D Oprisan G Maresca A Matsiota-Bernard P Guesdon JL Isolation of a specific DNA fragment and development of a PCR-based method for the detection of Mycobacterium genavense FEMS Immunol Med Microbiol 1999 23 3 243 252 10.1111/j.1574-695X.1999.tb01245.x 10219597

[B21] Albrecht H Rusch-Gerdes S Stellbrink HJ Greten H Jackle S Disseminated Mycobacterium genavense infection as a cause of pseudo-Whipple's disease and sclerosing cholangitis Clin Infect Dis 1997 25 3 742 743 10.1086/516941 9314476

[B22] Casanova JL Abel L Genetic dissection of immunity to mycobacteria: the human model Annu Rev Immunol 2002 20 581 620 10.1146/annurev.immunol.20.081501.125851 11861613

[B23] Matsiota-Bernard P Vilde F Nauciel C Mycobacterium genavense infection in normal and immunodeficient mice Microbes Infect 2000 2 6 575 580 10.1016/S1286-4579(00)00369-5 10884607

[B24] Catherinot E Lanternier F Bougnoux ME Lecuit M Couderc LJ Lortholary O Pneumocystis jirovecii Pneumonia Infect Dis Clin North Am 24 1 107 138 10.1016/j.idc.2009.10.010 20171548

[B25] Vrioni G Nauciel C Kerharo G Matsiota-Bernard P Treatment of disseminated Mycobacterium genavense infection in a murine model with ciprofloxacin, amikacin, ethambutol, clarithromycin and rifabutin J Antimicrob Chemother 1998 42 4 483 487 10.1093/jac/42.4.483 9818747

[B26] Griffith DE Aksamit T Brown-Elliott BA Catanzaro A Daley C Gordin F Holland SM Horsburgh R Huitt G Iademarco MF An official ATS/IDSA statement: diagnosis, treatment, and prevention of nontuberculous mycobacterial diseases Am J Respir Crit Care Med 2007 175 4 367 416 10.1164/rccm.200604-571ST 17277290

[B27] Ehlers S Richter E Gamma interferon is essential for clearing Mycobacterium genavense infection Infect Immun 2000 68 6 3720 3723 10.1128/IAI.68.6.3720-3723.2000 10816534 PMC97665

[B28] Kedzierska K Azzam R Ellery P Mak J Jaworowski A Crowe SM Defective phagocytosis by human monocyte/macrophages following HIV-1 infection: underlying mechanisms and modulation by adjunctive cytokine therapy J Clin Virol 2003 26 2 247 263 10.1016/S1386-6532(02)00123-3 12600656

